# Pulmonary arterial hypertension in a patient treated with dasatinib: a case report

**DOI:** 10.1186/s13256-017-1515-9

**Published:** 2017-12-29

**Authors:** Andris Skride, Matiss Sablinskis, Kristaps Sablinskis, Krista Lesina, Aivars Lejnieks, Sandra Lejniece

**Affiliations:** 1 Riga Stradins University, Riga, Latvia; 2 Pauls Stradins Clinical University Hospital, Riga, Latvia; 3 Riga East University Hospital, Riga, Latvia

**Keywords:** Pulmonary arterial hypertension, Dasatinib, Chronic myelogenous leukemia, Pleural effusion

## Abstract

**Background:**

There have been several reports on dasatinib-induced reversible pulmonary hypertension. This is the first reported case in Latvia; the patient did not discontinue the drug after the first adverse effects in the form of pleural effusions, which we speculate led only to partial reversion of the disease.

**Case presentation:**

A 67-year-old white man with chronic myelogenous leukemia was treated with the dual Src and BCR-ABL tyrosine kinase inhibitor dasatinib. After treatment with dasatinib he had multiple pleural effusions which were suspected to be caused by congestive heart failure. Later a transthoracic Doppler echocardiography and right-sided heart catheterization revealed severe pulmonary hypertension with pulmonary vascular resistance of 12 Wood units and mean pulmonary artery pressure of 53 mmHg. Computed tomography ruled out a possible pulmonary embolism; laboratory specific tests for human immunodeficiency virus, rheumatoid factor, and anti-nuclear antibodies were negative, and dasatinib-induced pulmonary arterial hypertension was diagnosed.

A follow-up right-sided heart catheterization and 6-minute walk test done a month after the discontinuation of dasatinib showed significant improvement: mean pulmonary artery pressure of 34 mmHg and pulmonary vascular resistance of 4 Wood units.

**Conclusions:**

Patients should always be closely monitored when using dasatinib for a prolonged time. Dasatinib-induced pulmonary hypertension may be fully reversible after the therapy is suspended, but the key factors involved are still unclear and need to be further studied.

## Background

Pulmonary hypertension (PH) is defined as an increase in mean pulmonary arterial pressure (mPAP) ≥ 25 mmHg at rest as assessed by right-sided heart catheterization (RHC). Normal mPAP at rest is 14 ± 3 mmHg with an upper limit of normal of approximately 20 mmHg.

The clinical classification of PH is intended to categorize multiple clinical conditions into five groups according to their similar clinical presentation, pathological findings, hemodynamic characteristics, and treatment strategy. Group 1, pulmonary arterial hypertension (PAH), includes precapillary PH, with a normal pulmonary capillary wedge pressure (PCWP) ≤ 15 mmHg, which is idiopathic, heritable, drug induced, or associated with various conditions; group 2 includes postcapillary PH associated with left-sided heart disease; group 3 corresponds to PH due to chronic lung diseases; group 4 corresponds to chronic thromboembolic PH; and group 5 consists of several forms of PH for which the pathogenesis is unclear or multifactorial [1].

Chronic myelogenous leukemia (CML) in the chronic phase (CP), a clonal myeloproliferative disorder, is caused by the constitutively active BCR-ABL tyrosine kinase resulting from the translocation that produces the Philadelphia (Ph) chromosome. Imatinib, an inhibitor of the BCR-ABL kinase (tyrosine kinase inhibitor, TKI), was the standard first-line therapy for patients with CML-CP for many years. Resistance to imatinib led to the development of novel TKIs such as dasatinib and nilotinib (second-generation BCR-ABL TKIs). Other TKIs with activity in CML include bosutinib and ponatinib [2–4]. Dasatinib therapy induces a complete cytogenetic response in approximately 50% of patients who do not have a response to imatinib or cannot tolerate it [5].

Compared with imatinib, dasatinib is associated with higher rates of pleural effusion, thrombocytopenia, and in rare cases PH, but lower rates of edema, gastrointestinal adverse events (AEs), musculoskeletal AEs, and rash [6]. The 2015 European Society of Cardiology guidelines for the diagnosis and treatment of PH includes dasatinib in the category of drugs that are “likely to induce pulmonary hypertension” [1].

There have been several reports on dasatinib-induced reversible PH [3]. This case is unique, as it is the first reported case in Latvia, and the patient did not discontinue the drug after the first adverse effects in the form of pleural effusions, which we speculate led only to partial reversion of the disease.

## Case presentation

Our patient is a 67-year-old white man, who works as a teacher in a small city. He does not smoke tobacco; he has a history of arterial hypertension grade I to II and coronary heart disease. His mother died from myocardial infarction due to occlusive coronary artery disease. He was diagnosed as having CML in 2006. The treatment was initially started with imatinib (Glivec, Novartis) 400 mg administered orally once daily. Imatinib 400 to 800 mg per day was taken for 53 months. He lost major molecular response (MMR) and imatinib therapy was replaced with dasatinib 100 mg orally administered once daily and after 6 months MMR was achieved. He was also using torasemide 20 mg orally administered once daily and metoprolol succinate 50 mg orally administered once daily. In April 2015 he developed increasing dyspnea on exertion, fatigue, and peripheral edema. He consulted his family physician, and a chest X-ray was done, confirming pleural effusion. On admission his heart rate was 97 beats per minute and blood pressure was 143/90 mmHg. Fever was not present. He presented with peripheral edema and diminished breath sounds. Pleural friction rub was present. Deformity of the spine accompanied by lower back pain was noted during neurological check-up. The pleural fluid was drawn out several times via thoracentesis (1.5 to 2 liters of exudate in total) but cytological analysis excluded malignancy, a GeneXpert® tuberculosis test of a bronchial smear was also negative, therefore, the pleural effusion was suspected to be caused by congestive heart failure. A complete blood count was normal, but his creatinine levels were elevated (Tables 1 and 2). Over the course of the next 4 months his general condition deteriorated as he experienced multiple recurrences of pleural effusion requiring drainage of the built-up fluid (Table 3). Dasatinib therapy was stopped in September 2015 after 42 months of treatment. A coronarography was done in September 2015; it did not reveal any hemodynamically important stenosis in his coronary arteries, thereby excluding coronary artery disease. An echocardiogram showed right ventricular dilation, estimated right ventricle systolic pressure of 125 mmHg, and severe tricuspid regurgitation suggesting PH.

**Table 1:** Complete blood count

Date	29 April 2015	19 February 2016
Erythrocytes, × 10^6^mm^3^	5.29	4.91
Leukocytes, × 10^3^mm^3^	3.66	4.3
Hemoglobin, g/dl	12.6	13.1
Hematocrit, %	38.8	41.5
Platelets, × 10^3^mm^3^	239	256
MCV, fL	84	82
MCH, pg	29.2	28.9
MCHC, g/dl	34.9	35.4
Neu, %	55.5	68.3
Ly, %	35.0	20.7
Eo, %	0.5	0.9
Ba, %	0	0
Mo, %	8.5	9.5

*Ba* basophils, *Eo* eosinophils, *Ly* lymphocytes, *MCH* mean corpuscular hemoglobin, *MCHC* mean corpuscular hemoglobin concentration, *MCV* mean corpuscular volume, Mo monocytes, NEU neutrophils

**Table 2:** Biochemistry of serum

Date	29 April 2015	19 February 2016
ALT, U/l	17.2	13.4
AST, U/l	5.0	3.3
Urea, mmol/l	14.7	12.2
Creatinine, μmol/l	187	87
Glucose, mmol/l	5.53	5.33
Total bilirubin, μmol/l	11.8	11.1
Direct bilirubin, μmol/l	5.6	5.2

*ALT* alanine aminotransferase, *AST* aspartate aminotransferase

**Table 3:** Biochemistry data of pleural fluid

Date	29 April 2015	08 May 2015	11 August 2015
Total serum protein, g/l	67.1	69.4	64.3
Serum LDH, U/l	303	296	320
Pleural fluid protein, g/l	29.5	28.1	32.7
Pleural fluid LDH, U/l	187	184	179
Glucose, mmol/l	6.42	6.24	7.47
Pleural fluid protein/Serum protein	0.43	0.4	0.51
Pleural fluid LDH/Serum LDH	0.62	0.62	0.55
Pleural fluid LDH > 2/3 of Serum LDH ULN	No	No	No

*LDH* lactate dehydrogenase, *ULN* upper limit of normal

RHC performed on 12 October 2015 revealed severe PAH with mPAP of 53 mmHg and normal left ventricle diastolic pressure (Table 4). A computed tomography scan confirmed the absence of pulmonary embolism; laboratory specific tests for HIV, rheumatoid factor, and anti-nuclear antibodies (ANA) were negative and dasatinib-induced PAH was diagnosed. The 6-minute walk test (6MWT) distance was limited to 165 m (Table 5). He started PAH-targeted treatment with sildenafil 20 mg × 3 orally administered and restarted CML therapy with imatinib 400 mg orally administered daily on 19 October 2015.

**Table 4:** Right-sided heart catheterization

Date	12 October 2015	16 November 2015
Parameter	(pre-intervention)	(post-intervention)
PAS, mmHg	84	55
PAD, mmHg	34	22
PAM, mmHg	53	34
CO, l/minute	4.07	5
CI, l/minute per m^2^	2.09	2.5
PVR, Wood units	12	4

*CI* cardiac index, *CO* cardiac output, *PAD* pulmonary artery diastolic pressure, *PAM* pulmonary artery mean pressure, *PAS* pulmonary artery systolic pressure, *PVR* pulmonary vascular resistance

**Table 5:** Six-minute walk test

Date	13 October 2015	16 November 2015
Distance, m	165	348

His condition rapidly improved, a check-up RHC done a month later showed mPAP of 34 mmHg, decreased pulmonary vascular resistance, and increased cardiac output values (Table 4). His 6MWT score was 2.1 times higher (Table 5). Echocardiography done in February 2016 revealed right ventricle systolic pressure of 50 mmHg; a complete blood count and biochemistry showed no abnormalities (Tables 1 and 2). He has been asymptomatic since, but treatment for PAH with sildenafil is still necessary. The last hematological check-up was in January 2017, he was still on imatinib 400 mg daily and had normal complete blood count and MMR (BRA-ABL was negative).

## Discussion

Our case provides insight on the chain of events that can occur in patients using long-term therapy with dasatinib, ultimately leading to partially reversible PAH. It raises awareness and adds data for future studies of this drug, as its usage and novel applications grow. Patients with CML in the European Union are mostly treated according to European LeukemiaNet recommendations, concerning the use of the five available TKIs, the evaluation of cytogenetic and molecular response, and the strategy of treatment [7]. Three TKIs (imatinib, nilotinib, dasatinib) are recommended first-line treatments.

Dasatinib is indicated for patients with newly diagnosed CML-CP or patients with CML in any phase who are resistant or intolerant to prior therapy [4, 7, 8]; this was the reason it was prescribed in our case. Each of the TKIs has typical AEs. Dasatinib often is associated with recurring pleural effusions, headaches, fatigue, dyspnea, and, in rare cases, PAH. It should also be noted that the therapy needs to be immediately suspended in patients with pleural effusions [9]. The connection between the recurring pleural effusions and dasatinib therapy was considered too late in our case, after our patient had gone through multiple hospitalizations, thoracenteses, and developed severe PH. His symptoms and overall condition greatly improved a month after discontinuation of the drug, confirming that dasatinib-induced PAH is reversible. Nevertheless a complete recovery was not observed, suggesting that some degree of pulmonary vascular remodeling had occurred. There have been reports of cases where a full recovery was achieved in patients with dasatinib-induced PAH but there is no information on whether the extent of recovery depends on the duration of therapy or individual risk factors.

The case presented is consistent with other reports of dasatinib-related PAH [3]. In all of these cases the patients were using dasatinib for a prolonged time (19 to 52 months), developed pleural effusions, and had a sudden onset of pulmonary vasculopathy and a rapid recovery after the discontinuation of the drug, which is not typical in patients with PAH. This leads to the conclusion that the pathophysiological mechanism differs from the typical vascular alterations seen in patients with PAH. Since the exact mechanism is still unknown, effective biomarker-based screening options and mechanism-based treatment are not available for patients with dasatinib-induced PAH. A better understanding of the pathophysiological mechanism would possibly create new treatment options in these patients, as well as increase our understanding of processes involved in the development of PAH.

## Conclusions

PAH is a serious and life-threatening adverse effect of long-term dasatinib use. Patients should always be closely monitored when using dasatinib for a prolonged time. Due to growing usage of dasatinib and novel applications in other diseases, overall awareness should be raised, as the risk factors involved in the development of PAH are still unclear. All cases should be reported to increase our knowledge, and new studies regarding the exact mechanisms should be conducted.

## Abbreviations

AE: Adverse event
ANA: Anti-nuclear antibodies
bpm: Beats per minute
CML: Chronic myelogenous leukemia
CP: Chronic phase
MMR: Major molecular response
mPAP: Mean pulmonary arterial pressure
PAH: Pulmonary arterial hypertension, PCWP, Pulmonary capillary wedge pressure
Ph: Philadelphia
PH: Pulmonary hypertension
RHC: Right-sided heart catheterization
TB: Tuberculosis
TKI: Tyrosine kinase inhibitor
6MWT: 6-Minute walk test
